# Description of the koala (*Phascolarctos cinereus*) brain based on magnetic resonance images

**DOI:** 10.3389/fneur.2026.1733030

**Published:** 2026-03-27

**Authors:** Kristy R. Q. Goh, Natasha B. Anthoniel, Timothy Stait-Gardner, Nyoman D. Kurniawan, Marianne D. Keller

**Affiliations:** 1 Sydney School of Veterinary Science, The University of Sydney, Camperdown, NSW, Australia; 2 Nanoscale Organisation and Dynamics Group, School of Science, Western Sydney University, Penrith, NSW, Australia; 3 Australian Institute for Bioengineering and Nanotechnology, Centre for Advanced Imaging, The University of Queensland, Brisbane, QLD, Australia

**Keywords:** brain mapping, diprotodontia, fasciculus aberrans, lissencephalic, marsupial, volumetry, structural MRI

## Abstract

**Introduction:**

Australia’s native fauna is unique through millions of years of isolated evolution resulting in a significant divergence of marsupial brain anatomy, and thus neural pathways, from eutherian mammals. This study provides a morphometric description of a mature female koala (*Phascolarctos cinereus*) through segmentation of high-resolution magnetic resonance images. As the availability of brain specimens of the endangered koala is scarce, brain segmentation has been performed on a single specimen.

**Methods:**

The brain of a naturally deceased koala was removed and fixed in 10% neutral buffered formaldehyde (pH 7.0). The MRI was acquired on a 9.4 T Bruker Biospec MR system with Paravision 7.0 software, using a 3D multigradient echo sequence, with TR/TE = 70 msec/ (8 echoes 4–32 msec), a field of view = 7.0 × 6.4 × 4.0 cm^3^. AMIRA post image processing software was used to create three-dimensional volume reconstruction of different brain structures, allowing for the calculation of their volumes.

**Results:**

The koala brain is lissencephalic and has a volume of 15720.98 mm^3^. It was found that the olfactory bulbs of the koala were 418 mm^3^ or 2.66% of the total brain volume (tbv). The cerebral hemispheres were 9336.92 mm^3^ (59.39% of the tbv). The interhemispheric connections were 148 mm^3^ (0.94% of tbv). The combined volume of the anterior commissure and fasciculus aberrans, which is specific to marsupials, has also been labeled and segmented. The ventricular system was 172 mm^3^ (1.09% of tbv). The brainstem was 1874 mm^3^ (11.92% of tbv). The cerebellum was 2304.69 mm^3^ (14.66% of tbv).

**Conclusion:**

This study can be used as a basis for clinical imaging of koalas and as baseline data for future research into brain development of koalas and other marsupials.

## 1 Introduction

Marsupials have been identified as ideal candidates for pursuing further research on cortical evolutionary development, knowing that similarities in the nervous system between marsupials and placental mammals arise due to convergence (1). Despite this, there have been few studies of the brain anatomy of one of the most familiar and high-profile marsupials, the koala (*Phascolarctos cinereus*). The last paper on magnetic resonance imaging (MRI) of the koala brain, which only focused on basic brain morphology, vascularisation and encephalization, appeared almost two decades ago (2). There are limited data on the histology of the koala brains, with the sole resource being four stained histological sections through the cerebral hemisphere of an adult koala from the Nelson Brain Collection (3). There are some recent brain morphometric studies performed on other marsupials such as the quokka (*Setonix brachyurus*) and the tammar wallaby (*Macropus eugenii*) (4, 5). However, while both koalas and macropods are part of the order Diprotodontia, macropods are in the family Macropodidae, part of the suborder Phalangerida. On the other hand, the koala belongs to the family Phascolarctidae, which is closely related to the wombats (family Vombatidae), both koalas and wombats are within the suborder Vombatiformes, and hence only distantly related to macropods. The lack of a koala brain atlas limits our ability to understand evolutionary brain development of the koala and also makes it difficult to diagnose anatomical abnormalities in koala brains in clinical settings.

Segmentation of histological images has been used to develop brain atlases of other species such as rats (6). Segmentation of magnetic resonance (MR) images into anatomical regions will support the production of a koala brain atlas by providing brain morphometric features and allow volume measurements to be obtained for gray and white matter of the cerebral cortex, as well as for the individual regions of the brain. This will allow comparisons to be made with other species and lay the groundwork for further refinement in the delineations of different sections of the brain in koala brain research by aiding in the identification of landmarks for the development of cortical test points in the production of koala brain template images, which will be the fundamental step toward a koala brain atlas (7).

Interhemispheric connections in marsupials are of interest as previous studies have shown that marsupials do not have a corpus callosum but have an additional tract, the fasciculus aberrans (4). In the tammar wallaby, the fasciculus aberrans is just dorsal to the anterior commissure, which is also known as the rostral commissure (4). While there have been other papers which included drawings or diagrams showing the location of the fasciculus aberrans, there have been no other studies clearly delineating this structure on an actual koala brain (8). Our study aims to provide more information on the volume of the interhemispheric connections.

The overall goal of this study is to provide a descriptive and comparative neuroanatomical study of the adult koala brain by segmentation of a three-dimensional MR datasets and measuring the volumes of the different structures of the adult koala brain as well as the volume of the white matter in the cerebral hemispheres of the brain.

## 2 Materials and methods

A deceased adult female koala of unknown age was presented to the University Veterinary Teaching Hospital Sydney (UVTHS). Necropsy was performed by a veterinary pathologist with the primary goal of determining the cause of death of the Koala. To be able to collect marsupials for imaging purposes under the Environmental Protection Agency NSW Scientific License SL102840 only animals that died of causes unrelated to our research could be collected. No animal ethics was required.

During necropsy, the brain was removed and fixed in 10% neutral buffered formaldehyde (pH 7.0) for 2 months. Subsequently, the brain was washed in saline to improve contrast before imaging.

### 2.1 MRI sample preparation and image acquisition

Prior to MRI, the brain sample was incubated with 0.1% v/v of Magnevist (Bayer, Germany) in saline for 1 week at 4 ^o^C (9). High resolution anatomical imaging was performed in saline using a 9.4 T Bruker Biospec MRI located at the Center of Advanced Imaging, University of Queensland. The system is a 300 mm ultra shield refrigerated 30 cm magnet interfaced to a Bruker Avance III spectrometer (Bruker Biospin Ettlingen, Germany). Paravision 7.0 software was used for acquisition and reconstruction (Bruker Biospin, Ettlingen, Germany).

MR images with a combination of T1 weighting and multiple T2* weighting (T1/T2*-weighting) were used to scan the adult koala brain. The MRI scan parameters were TR/TE = 70 ms/ (8 echoes 4–32 ms); the field of view was 7.0×6.4x4cm with a single acquisition and without repetitions, with an imaging time of 1 h 14 min 40 s. MR images were zero-filled in the phase dimensions by a factor of 1.5 to produce images at 133 micrometer isotropic 3D resolution.

A 3D diffusion weighted imaging (DWI) dataset was also acquired using a Stajeskal-Tanner (DWI spin-echo) sequence with the following parameters: TR/TE = 150/20 ms, *δ*/*Δ* = 4/10 ms, 3 b = 0 and 30 diffusion-encoding directions with b = 3,000 s/mm^2^ and acquisition time of 15 h 28 min.

### 2.2 Processing of anatomical imaging data sets and delineation

Brain segmentation was carried out with reference to the koala brain histological sections, the tammar wallaby stereotaxic brain atlas and the magnetic resonance reconstruction of the quokka brain (4, 5). Terminology used in this study was based on those used in previous studies on marsupial brain anatomy, though the Nomina Anatomica Veterinaria (NAV) terms^1^ were also included to remain congruous with veterinary anatomy literature.

AMIRA (version 6) software (Thermo Fisher, Massachusetts, United States) was used for structure delineation and labeling (10).

**Figure 1:**
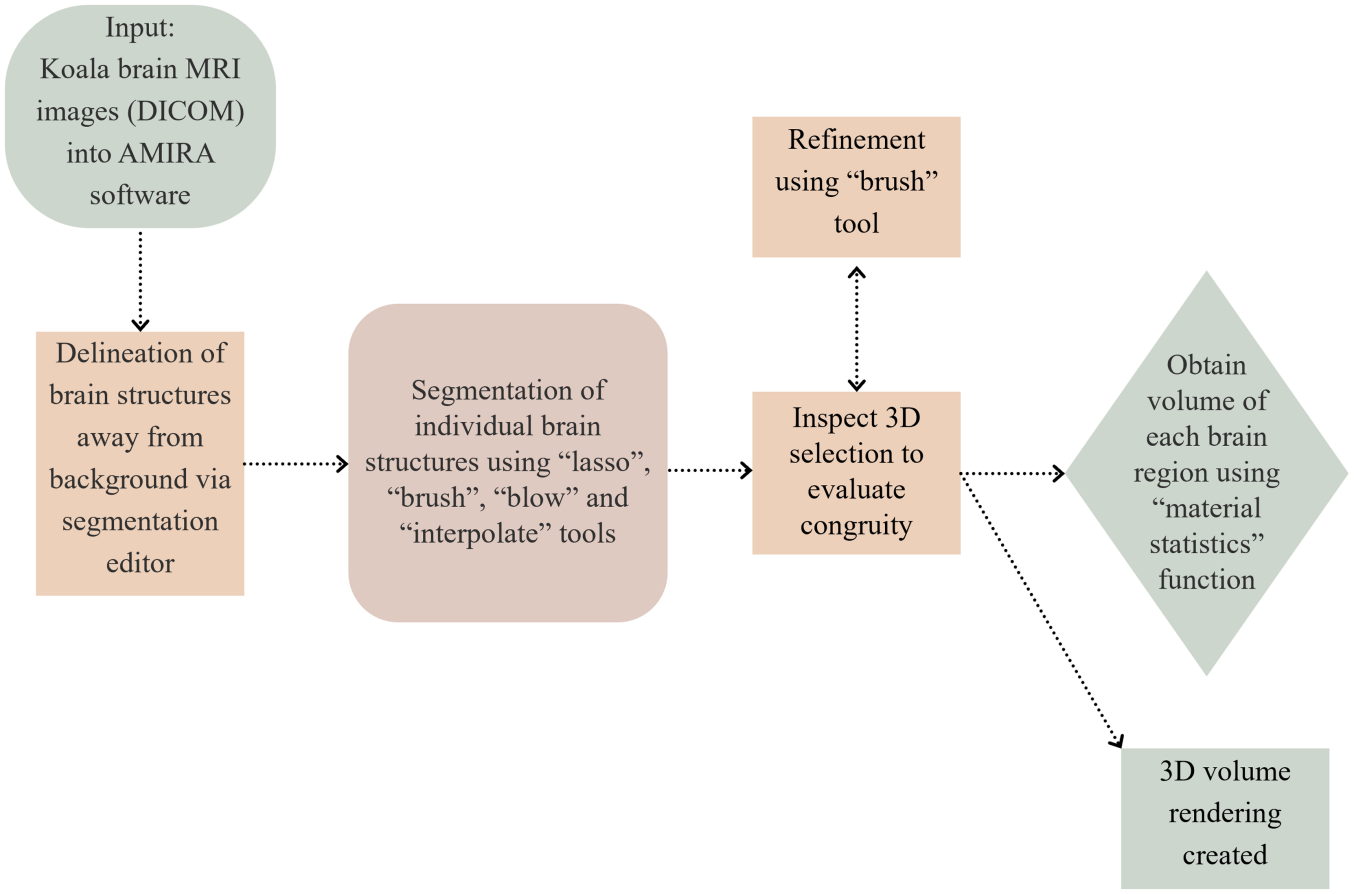
Illustration of the segmentation process.

Upon loading the MRI scan into AMIRA, the segmentation editor was used to delineate the margins of the brain structures away from the background and the interior assigned to a component labeled “interior.” This was conducted by using the “lasso” tool which allows drawing of a free-handed outline of the brain section, followed by the “brush” and “blow” tool to select areas which are missed by the “lasso” tool (Figure 1). The “interpolate” function was also used to generate a congruous brain region across narrow bands of slices (3–25, depending on complexity of brain structures). The resulting 3D selection was then inspected and, if necessary, corrected using the brush tool. Within the “interior” segment, various structures of the brain were segmented and assigned to different brain regions. After a particular region of the brain had been segmented completely, the component was locked such that there would be no changes to the mapped region, preventing overlapping segmentation.

**Figure 2:**
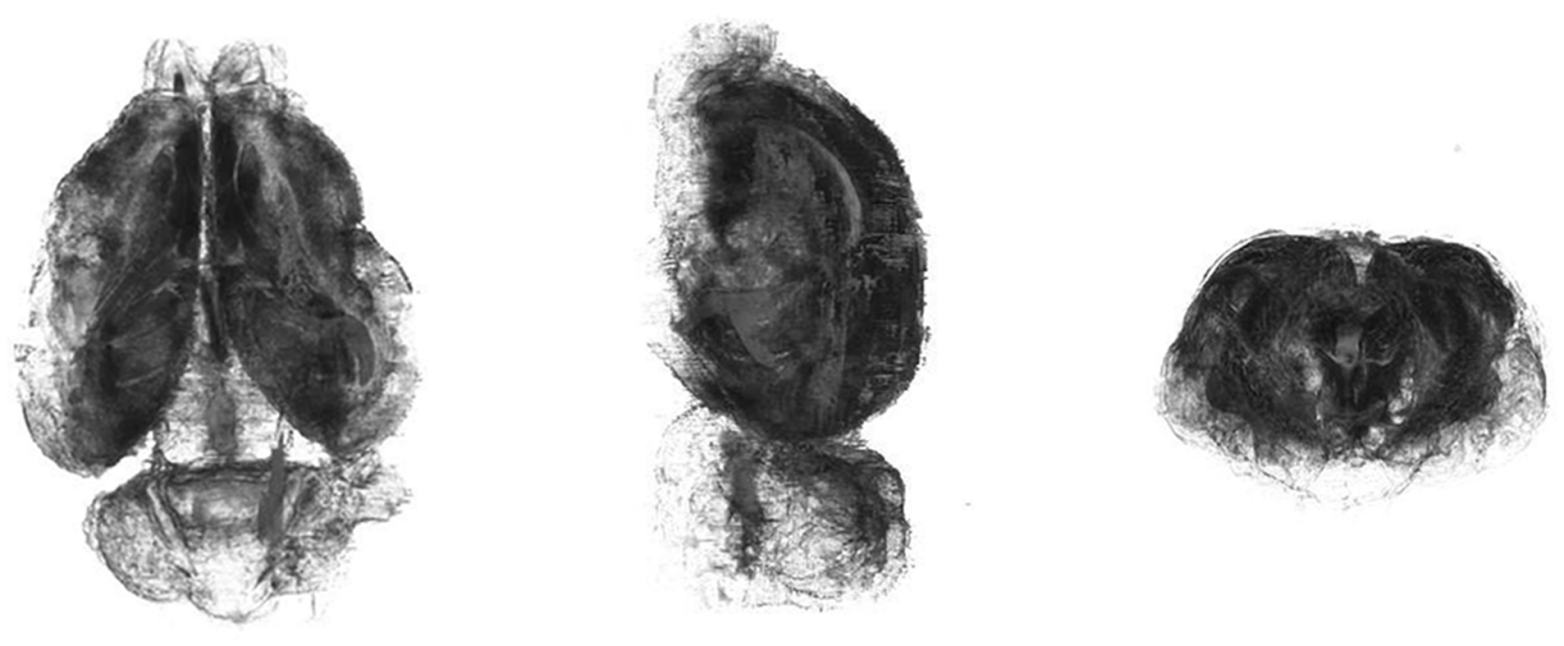
3D rendering of the brain of the koala, viewed in the coronal, sagittal, and axial planes, respectively, from left to right.

In order to obtain the volume of the different segments, the “material statistics” function within AMIRA was utilized (10). Volumes of the segmented regions of the brain were computed based on voxel count, which was done automatically by AMIRA based on the voxel dimensions obtained from the metadata in the DICOM files (Table 1). A 3D rendering of the adult koala brain was created (Figure 2). 3D rendering is a 3D visualization technique that displays complex 3D MRI data, by casting virtual light rays through the data volume, integrating opacity from each point (voxel) along the ray to create a continuous, realistic 3D image, revealing internal structures, allowing users to see inside objects, as well as the thickness of the object and thus enable understanding of complex anatomical relationships.

**Table 1:** Segmentation of Koala brain using T1/T2* weighted images.

Structure	Volume (mm^3^)	Proportion of brain (%)
Olfactory Bulbs	418.21	2.66
Cerebrum	370.81 (hippocampus) + 24.38 (internal capsule) + 23.82 (external capsule) + 8917.91(remaining section of the cerebral hemispheres) = 9336.92	59.39
Amygdala	1.01	0.01
Internal Capsules	24.38	0.16
External Capsules	23.82	0.15
Hippocampus	370.81	2.36
Interhemispheric connections	124.45 (ventral hippocampal commissure) + 23.70 (anterior commissure + fasciculus aberrans) = 148.15	0.94
Ventral Hippocampal Commissure	23.70	0.15
Anterior Commissure + Fasciculus aberrans	124.45	0.79
Ventricular system	172.31	1.10
Olfactory Ventricles	7.54	0.05
Lateral Ventricles	110.21	0.70
Putamen	30.38	0.19
Midbrain	646.78	4.11
Pons	702.44	4.47
Thalamus	412.84	2.62
Third Ventricle + Cerebral/Mesencephalic Aqueduct	23.39	0.15
Medulla	525.51	3.34
Fourth Ventricle	31.17	0.20
Cerebellum	2304.69	14.66
Caudate Nucleus	110.70	0.70
White matter of cerebral hemispheres	1495.47	9.51

Numerical summarization of volumetric and percentage of the brain results per region including the olfactory bulbs, cerebral hemispheres, internal capsules, external capsules, hippocampus, amygdala, interhemispheric connections, ventral hippocampal commissure, anterior commissure + fasciculus aberrans, ventricular system, olfactory ventricles, lateral ventricles, putamen, midbrain, pons, thalamus, third ventricle + cerebral/mesencephalic aqueduct, medulla, fourth ventricle, cerebellum, caudate nucleus, white matter of cerebral hemispheres, remaining unsegmented parts of brain.

The MR image with both T1/T2*-weighting (TE = 24 ms) was chosen for image segmentation as it provided the best image contrast compared to the other TEs. The approach in this study is different to that of Kariyappa et al. (5), as imaging was performed in saline with 0.1% magnevist without using Fomblin. Air bubbles on the surface and interior of the brain were difficult to remove prior to the scan. To produce the images in this manuscript, areas outside of the brain were removed by masking non-brain areas. The brain mask was derived by averaging all of the 30 diffusion-weighted images, such that signals from the surrounding buffer were eliminated, leaving a brain only signal.

There was an artifact in the specimen which spanned the left side of the cerebral hemisphere. This issue was mitigated by delineating the outline of the artifact and ensuring that it is not classed as the interior of the brain. Additionally, a section of the cerebellum on the left side was incongruous with the majority of the cerebellum, indicating a section of cerebellum was likely lost during processing. Impact of this lost section of cerebellum was limited as the cerebellum is a largely symmetrical structure and labeling could occur on the right side. These artifacts were taken into account by deducting the volume occupied by these gaps from the overall brain volume.

Existing papers (11, 12) did not clearly demarcate the start and end of the internal and external capsules, so in this study, the beginning of the dorsal anterior commissure and fasciculus aberrans was defined when the two capsules rejoined after being separated by the putamen.

With regards to the interhemispheric connections, this study has followed the demarcation as stated in the Tammar Wallaby atlas which considered the continuation of white matter tracts from the internal and external capsule as the deep cerebral white matter.

At these levels where the border between the caudate nucleus and the hippocampus were obscured by the cut made during necropsy, symmetry of the brain from the unaffected side was relied upon with the side unaffected side by the artifact used to decide the demarcation between hippocampus and caudate nucleus.

Segmentation of the brainstem was conducted based on the understanding that the midbrain connects the pons and diencephalon, while the pons connects the medulla oblongata to the midbrain (13).

The formula for calculating cerebellar quotient used for comparing relationship between brain mass and cerebellar volume is adapted from Maseko et al. (14), which is CQ = Cb_vol_/(0.145 x M_b_^0.978^), whereby CQ is cerebellar quotient, Cb_vol_ is cerebellar volume and M_b_ is brain mass.

## 3 Results

**Figure 3:**
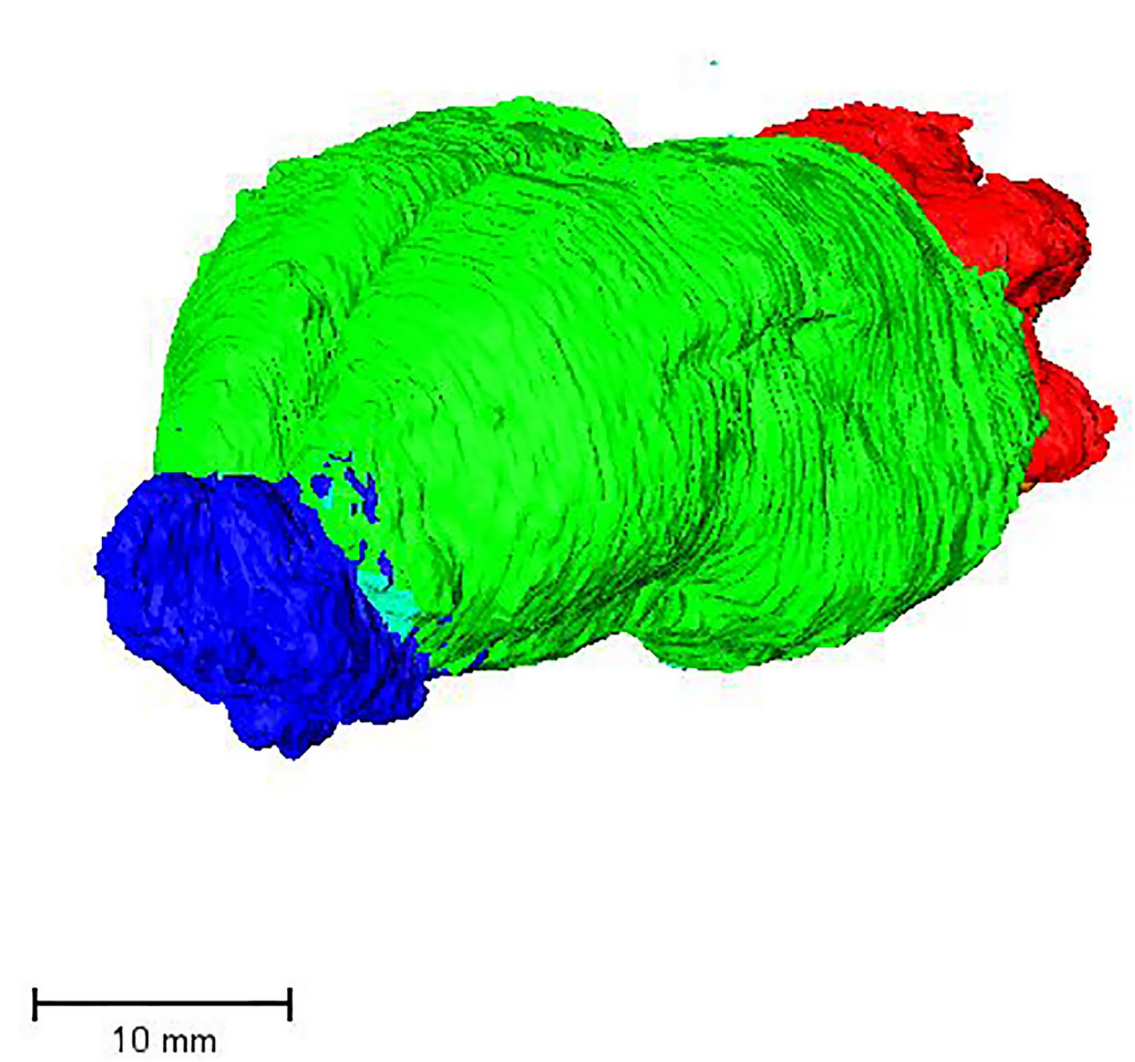
Surface rendering of the olfactory bulbs (blue), cerebrum (green), and cerebellum (red).

**Figure 4:**
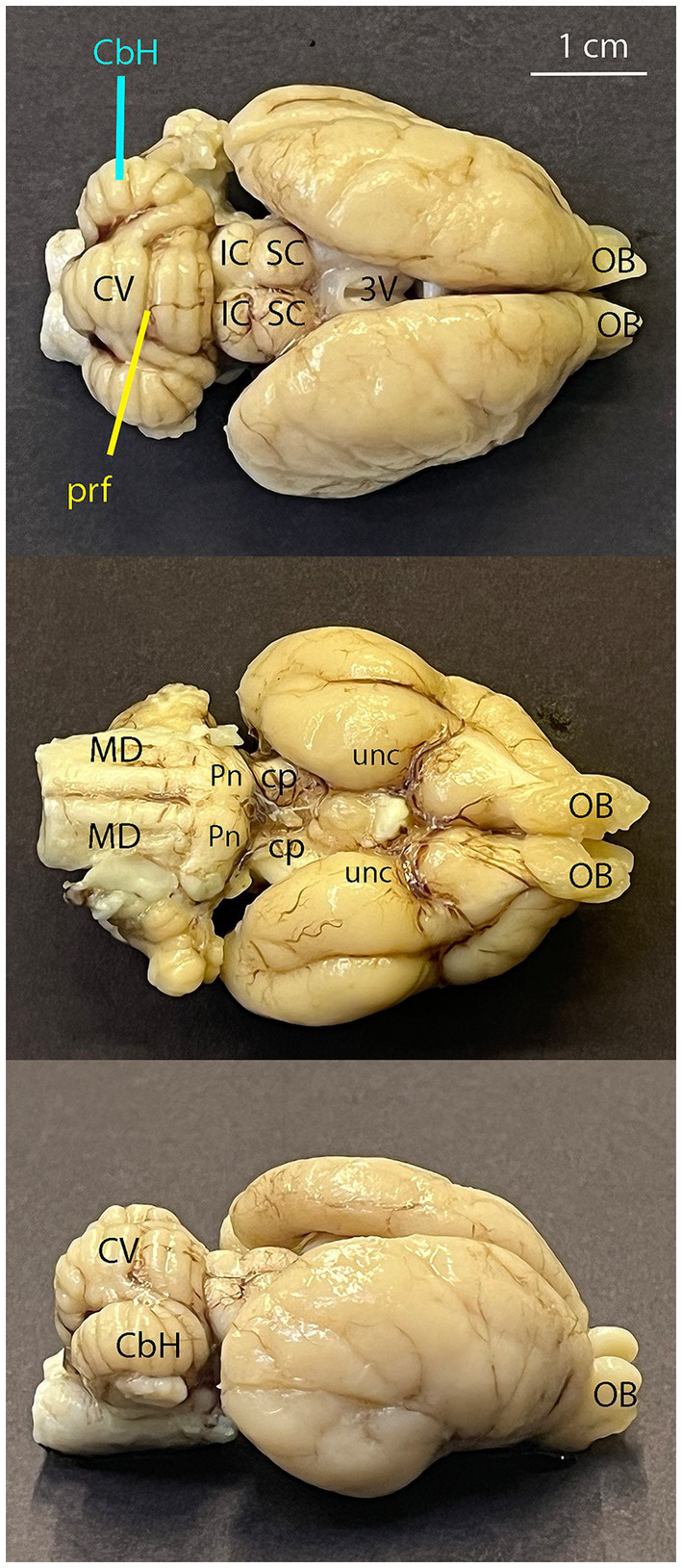
Photos of the Koala brain, top = dorsal view, middle = ventral view, bottom = laterodorsal view. CbH, cerebellar hemisphere; prf, primary fissure of the cerebellum; CV, cerebellar vermis; MD, medulla oblongata; Pn, pontine nuclei (pons); cp, cerebellar peduncle; IC, inferior colliculus; SC, superior colliculus; unc, unculus; 3 V, third ventricle; OB, olfactory bulb.

In the dorsal view, the cerebral hemispheres have an ovoid outline, with olfactory bulbs taking up one-ninth of the length of the entire koala brain (Figures 2, 3). The cerebellar cortex is lissencephalic and the cerebellum is foliated (Figure 4).

The volume of the entire adult koala brain segmented in this study was 15720.98 mm^3^ (Figures 2, 4).

### 3.1 Sectional anatomy of the koala brain

**Figure 5:**
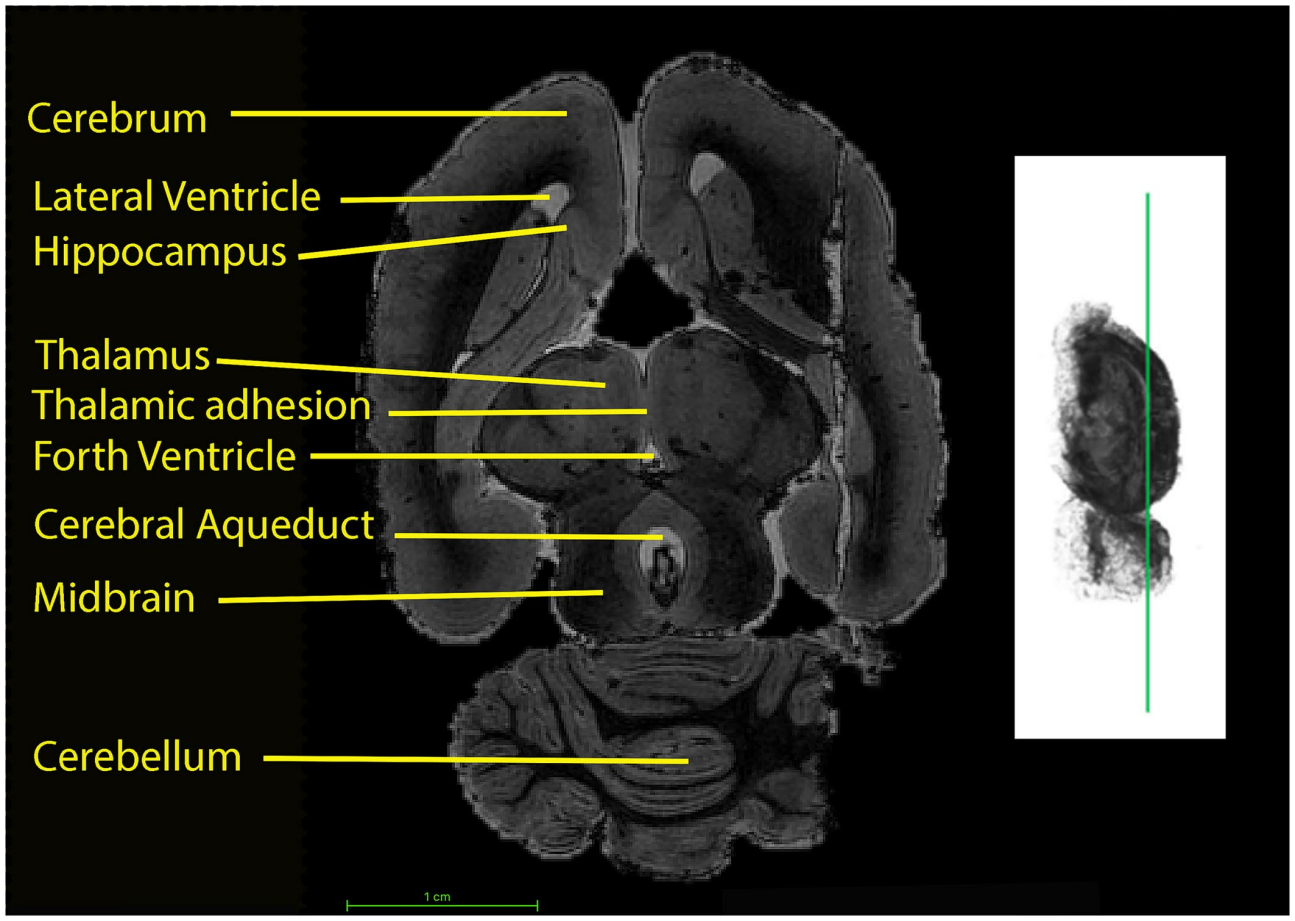
On the left: Horizontal section through the brain of the koala. Sections from the cerebrum at the top to the beginning of the spinal cord segment at the bottom of the figure are shown. The red line superimposed on the 3D rendering (right) marks the anatomical plane corresponding to the slice visualized on the left.

**Figure 6:**
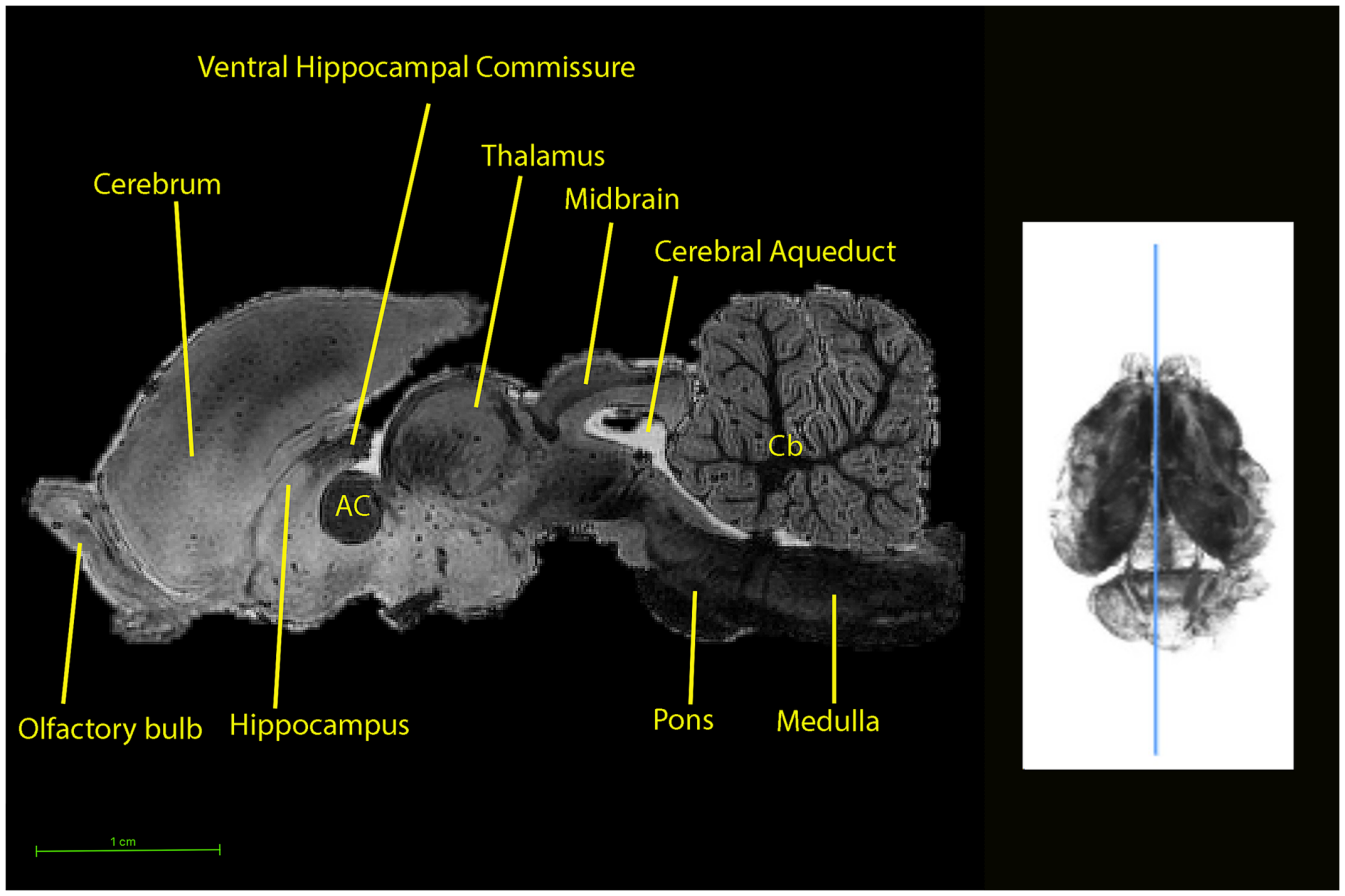
Sagittal midline sections through the brain of the koala (left) at the level indicated by the line on the 3D rendering using a T1w scan (right). Sections from the olfactory bulb on the left to the beginning of the spinal cord segment on the right are shown. AC, Anterior commissure and fasciculus aberrans; Cb, cerebellum.

The following sections are divided into the different anatomical regions of the koala brain. Descriptions of the major findings of each anatomical region as revealed by T1/T2*w images are presented in rostrocaudal sequence (Figures 5, 6).

#### 3.1.1 Olfactory bulb

**Figure 7:**
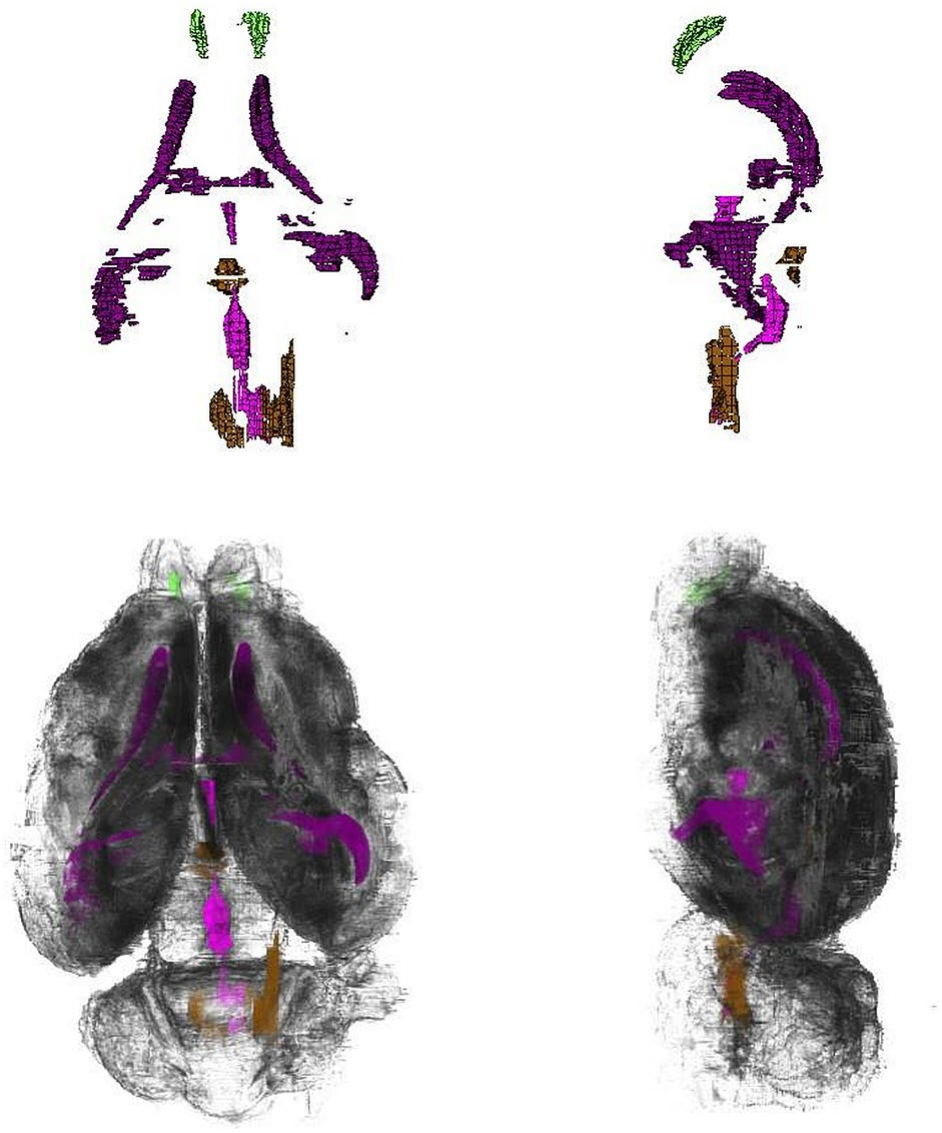
Top row from left to right: Three-dimensional reconstruction of the ventricular system, showing the horizontal and sagittal plane; bottom row from left to right: 3D rendered reconstruction with the ventricular overlay, viewed in the horizontal and sagittal plane (key: Green = olfactory ventricle, Purple = lateral ventricle, Pink = third ventricle joining with mesencephalic aqueduct, Brown = fourth ventricle).

The olfactory bulb (Figures 3, 4, 6) is the most rostral distinct structure separated from the cerebral hemispheres, and it contains a central cavity, the olfactory ventricle (Figure 7).

The olfactory bulb is structurally made up of distinct concentric layers and is connected to the cerebral cortex through the olfactory peduncles. There was close to no difference in signal intensity between the olfactory bulb and olfactory peduncle, hence the olfactory peduncle was not differentiated from the olfactory bulb in this study.

Using the stereotaxic atlas of the brain of the Tammar Wallaby as a reference, we were able to distinguish the olfactory ventricle and the olfactory nerve layer of the olfactory bulb during segmentation but not the other layers due to poor resolution (4). The olfactory bulb was recorded to be approximately 418.21 mm^3^ (Table 1), which makes up 2.66% of the total brain volume. The volume of the right and left olfactory bulbs combined was calculated, to ensure standardization in calculating brain regions and brain ratios.

#### 3.1.2 Cerebrum, cerebral hemispheres

The white matter within each hemisphere surrounds each lateral ventricle, respectively, (Figures 5, 7).

The cerebral hemispheres account for a large proportion of the brain’s total volume (Table 1). This volume encompasses major structures such as the hippocampus, internal capsule, and external capsule. While the cerebrum takes up a substantial proportion of the total brain volume, the cerebellum is the next largest structure.

##### 3.1.2.1 Interhemispheric connections

**Figure 8:**
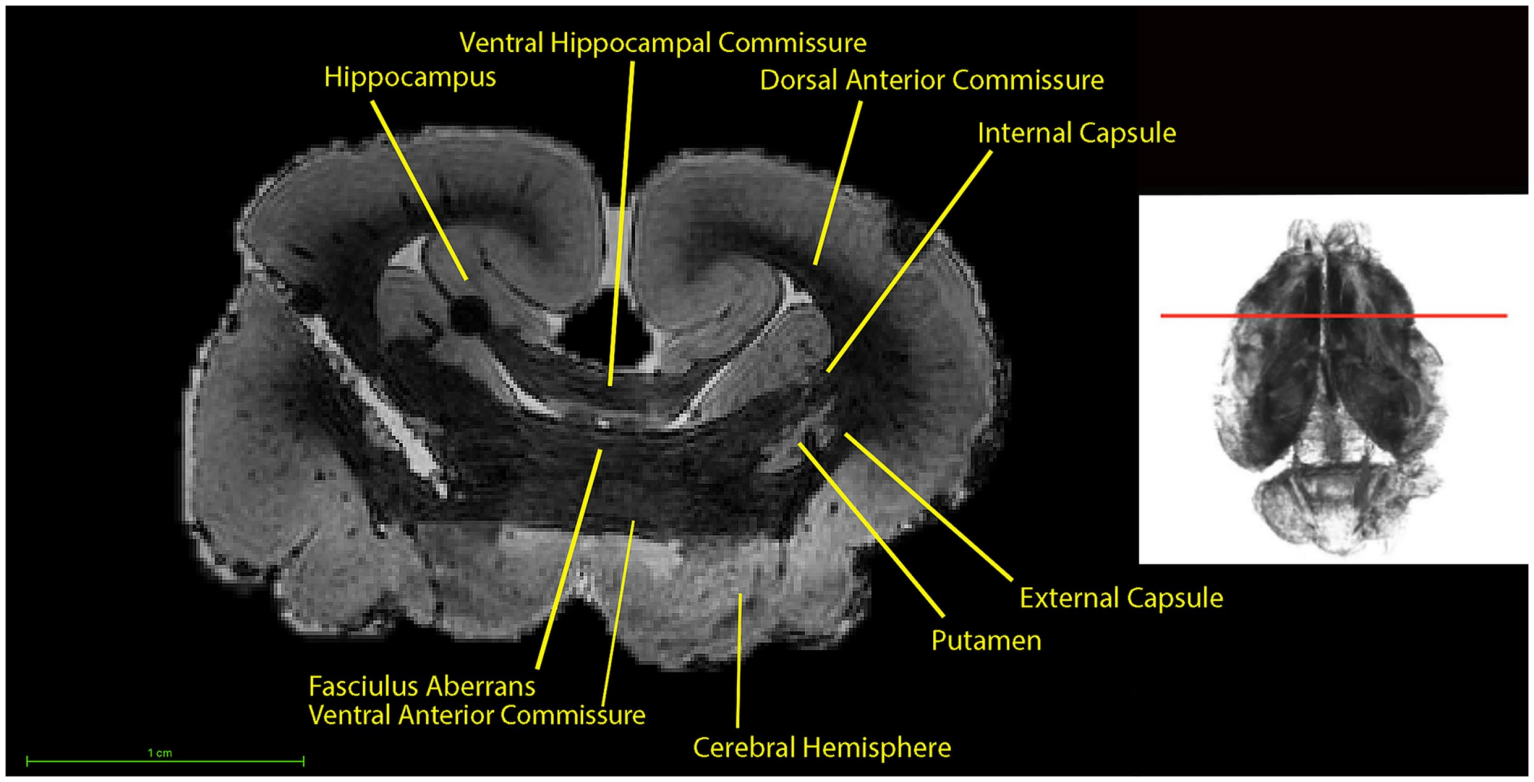
Coronal section (left), at the level indicated by the line on the 3D rendering using T1w scan (right). Top of the image is the dorsal part of the brain. The fasciculus aberrans could not be clearly distinguished from the anterior commissure as there was no gray matter separating the two, causing the fasciculus aberrans and the anterior commissure to appear as a single large bundle of white matter tracts.

The interhemispheric connections (Figure 8) demarcated on the scan include the anterior commissure and the ventral hippocampal commissure. The fasciculus aberrans is also an interhemispheric connection and continues on from the internal capsule, located immediately dorsal to the anterior commissure (Figure 8). The fasciculus aberrans cannot be easily differentiated from the anterior commissure, as there was no gray matter separating the two, causing the fasciculus aberrans and the ventral anterior commissure to appear as a single large bundle of white matter tracts (Figure 8). Therefore, the two structures were segmented as one. The projection fibers consist of the internal capsule and the external capsule, which send impulses down from each side of the telencephalon down to the brainstem (Figure 8).

The putamen serves as the landmark whereby the anterior commissure joins with the external capsule. The internal capsule is located between the caudate nuclei and the putamen, while the external capsule is located between the putamen and the externally located gray matter. The ventral hippocampal commissure is located medioventrally to the hippocampus (Figure 8). The dorsal anterior commissure is located lateral and dorsolaterally to the hippocampus and is itself surrounded laterally by cerebrum (Figure 8).

##### 3.1.2.2 Hippocampus

**Figure 9:**
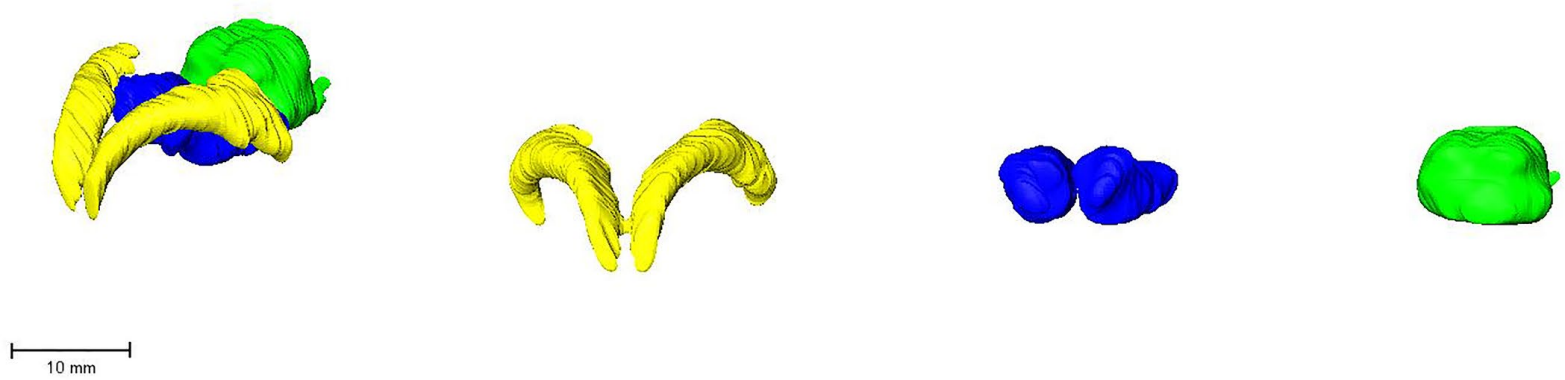
Surface rendering of hippocampus (yellow), thalamus (blue), and midbrain (green). Left lateroventral view of all three structures, followed by a craniolateral view of the individual structures.

**Figure 10:**
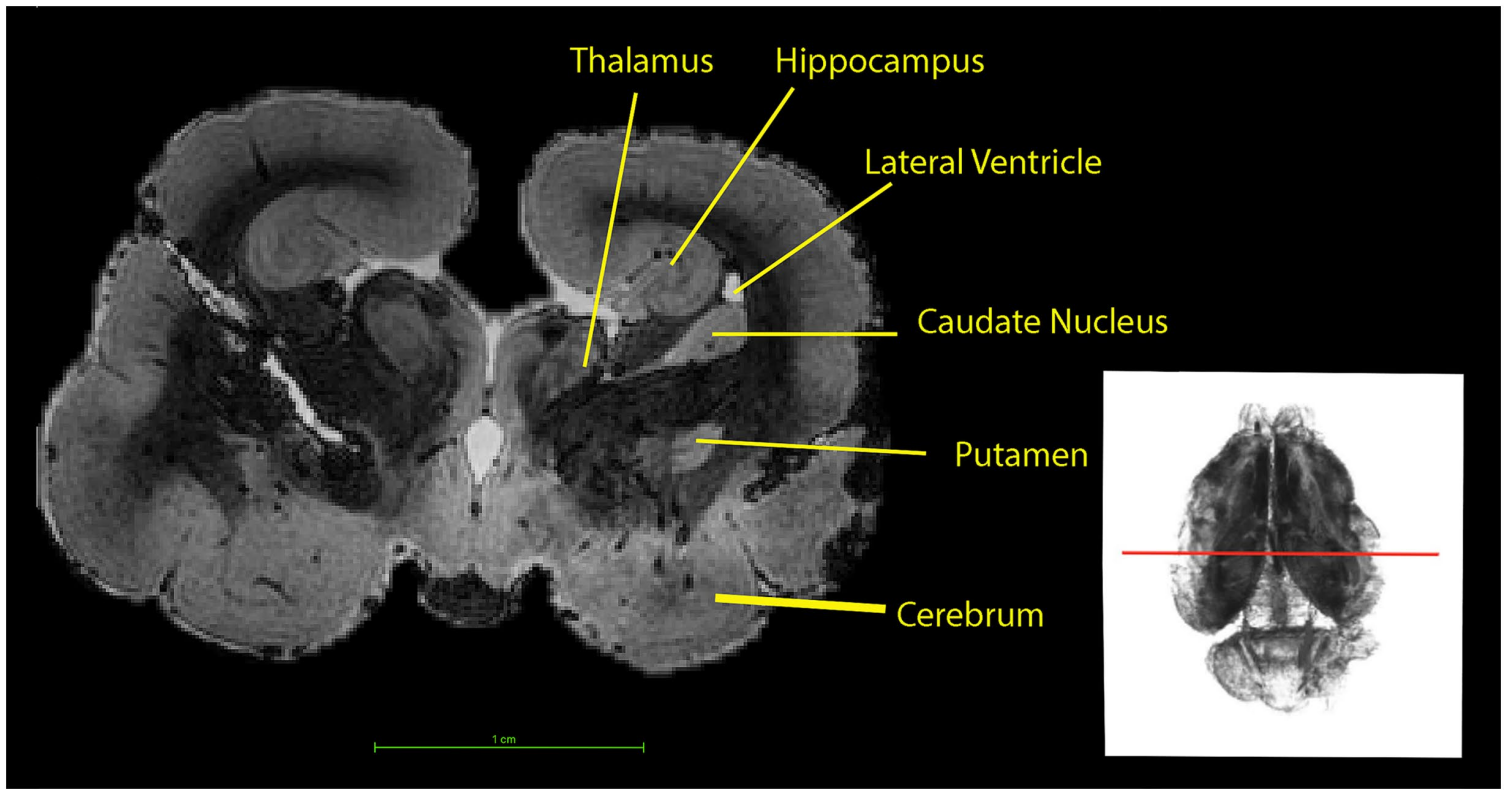
Coronal section (left) at the level indicated by the line on the 3D rendering using T1w scan (right), further caudally as compared to section in Figure 4. The top of the image is the dorsal part of the brain.

The hippocampus (Figure 9) is easily distinguishable from the surrounding tissues due to the difference in signal intensity highlighting the inward curving nature of the stratum pyramidale (Figures 5, 9, 10). The stratum pyramidale and stratum granulosum of the dentate gyrus can be identified in the hippocampus due to the significant difference in signal intensities of these two structures from the rest of the hippocampus. The ventral hippocampal commissure, a bundle of nerve fibers connecting the left and right hippocampi across the midline of the brain (Figure 8).

#### 3.1.3 Putamen, caudate nucleus and amygdala

The putamen lies between the internal and external capsule (Figures 8, 10). The caudate nucleus was identified as being between the lateral ventricle and the internal capsule on the coronal section (Figure 10). The uncus as the surface projection of the almond-shaped amygdala is visible in the ventral view of the brain (Figure 4).

#### 3.1.4 Thalamus

The thalamus (Figures 2, 3) makes up the core of the diencephalon with its generally rounded appearance surrounded by the epithalamus, subthalamus, hypothalamus and metathalamus. The thalamus showed lighter signal intensities than its surrounding structures on the scan. The left and right thalamus are joined by the interthalamic adhesion (Figure 9), which is surrounded by the third ventricle (Figure 10).

#### 3.1.5 Midbrain, pons and medulla

**Figure 11:**
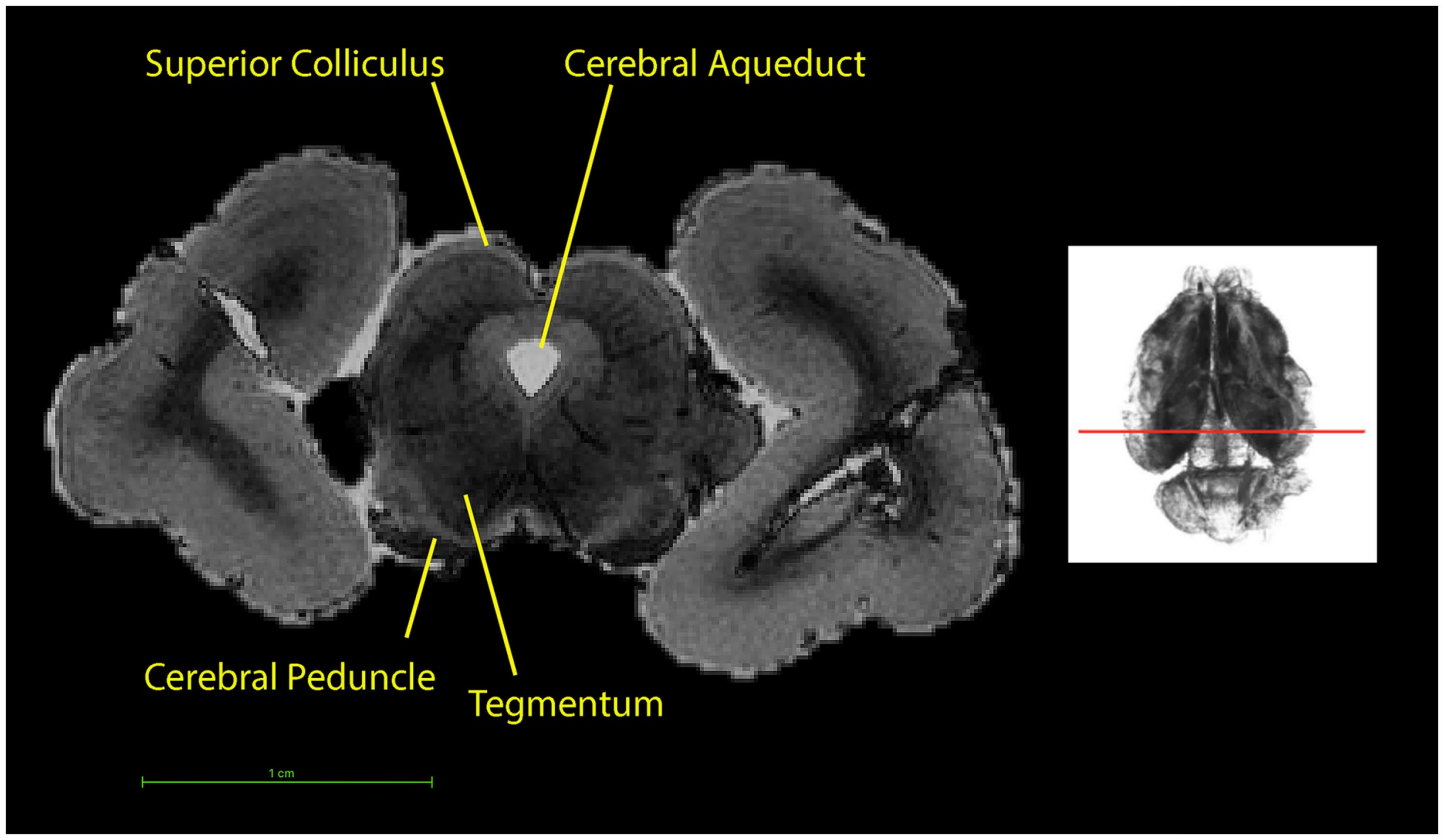
Coronal section (left) at the level indicated by the line on the 3D rendering using T1w scan (right). The top of the image is the dorsal part of the brain.

The midbrain (Figure 9) mapped in this study includes adjacent parts of the diencephalon and metencephalon since these structures cannot be differentiated on the T1w scan. The superior colliculus is located in the caudal part of the midbrain (Figure 4), while the tegmentum forms the floor of the midbrain surrounding the mesencephalic aqueduct. The cerebral peduncle is the connection between the midbrain and the cerebellum dorsally. Structures such as the superior colliculus, tegmentum and cerebral peduncle have been mapped based on their relative positions to the cerebral aqueduct as well as their signal intensities. In this study the tegmentum had the darkest signal intensity, followed by the superior colliculus and the cerebral peduncle having the lightest signal intensity (Figure 11). The surface projections of the superior and inferior colliculus and the cerebellar peduncle can be seen in the ventral view of the brain (Figure 4).

The pons has been mapped based on it being located between the midbrain and the medulla and ventral to the cranial half of the cerebellum (Figures 4, 6). Differentiation of pons and mesencephalon was difficult due to similar light intensity; therefore, the pons segmented in this study includes some aspects of the mesencephalon.

#### 3.1.6 Cerebellum

The cerebellum is located dorsal to the brainstem, the medulla and the pons (Figures 4, 6). The cerebellum has well-developed, numerous, small, parallel folds called folia covering the cerebellar surface, increasing its surface area, and fissures - deep grooves separating these folia as well as sulci dividing the medial vermis from the lateral hemispheres (Figure 4). The cerebellar peduncles (Figure 4) which are located rostral to the mesencephalon, middle to the pons and caudal largely to the myelencephalon are mapped as part of the cerebellum in this study.

The volume of the cerebellum was approximately 2304.69 mm^3^ (Table 1), which is 14.66% of the total brain volume.

#### 3.1.7 Ventricles

The ventricular system (Figure 7) is made up of the olfactory ventricle, lateral ventricle (Figure 11), third ventricle (Figure 4), fourth ventricle (Figure 5) and the cerebral/mesencephalic aqueduct (Figures 5, 6, 11). The olfactory ventricle is the cavity within the olfactory bulb while the lateral ventricle is within the cerebral hemisphere. The third ventricle is located between the right and left thalamus, while the fourth ventricle is the cavity which is dorsal to the pons and medulla and ventral to the cerebellum. The third ventricle and fourth ventricle are connected to each other via the cerebral aqueduct.

The volume of the ventricles measured in this study was 172.31 mm^3^ (Table 1).

## 4 Discussion

The present study uses MRI technology to segment the brain of a diprotodontian marsupial, the koala (*Phascolarctos cinereus*) and compares it to published knowledge of other marsupials and eutherians. A brain atlas is crucial for appreciating the basic brain structure in diprotodontids and to understand how the brains of diprotodontians differ from other marsupials and eutherians.

### 4.1 Investigation methods: benefits and limitations

This study comprises high resolution structural data, elucidating brain regions in the koala. T1/T2* weighted MRI images provide a novel way of observing the structures of the koala brain in three dimensions without the need for animal ethics approval. While histological sections have the highest resolution and therefore enable easier differentiation of cell layers, it is difficult to obtain an accurate reconstruction of the brain due to the distortions created in the sectioning process, stitching artifacts and possible non-standard angles during sectioning (15).

### 4.2 General anatomical findings

#### 4.2.1 Volume and morphological features of brain

The volume of the koala brain used in this study was approximately 15 mL (Table 1), which is lower than the single previously reported value for volume of the koala brain (20 mL) (16). The volume recorded in this study included the volume occupied by the ventricles, while in the study conducted by (16), the method of measuring volume was not specified, and it is unclear whether ventricular volume was taken into account. It should also be noted that there is intra-species variation in brain sizes with stepwise changes in brain–body allometry through ontogeny across many different species including the eastern gray kangaroo (17).

#### 4.2.2 Olfactory bulb

The koala has been reported to have three layers in the olfactory bulb, with the olfactory nerve layer and neuroepithelium separated by a layer in between (18). The koala has this in common with the tammar wallaby and brushtail possum, making the tammar wallaby a good reference for segmenting the olfactory bulb. The mouse which also has a lissencephalic brain has a more differentiated olfactory bulb consisting of six layers: olfactory nerve layer, glomerular layer, external plexiform layer, mitral cell layer, internal plexiform layer and the granule cell layer (19).

The olfactory bulb measured approximately 418.21 mm^3^ (Table 1), representing 2.66% of the total brain volume. This proportion is lower than that reported for the brush-tailed possum and the quokka, in which the olfactory bulbs comprise 3.1 and 3.4% of total brain weight, respectively (4).

The higher proportion of olfactory bulb weight in brush-tailed possum and quokka was attributed to the poorer development of the cerebellum, midbrain, striatum and neocortex. Research on olfactory communication in koalas highlights the prominent sternal gland which adult male koalas often use to mark their scent on the base of trees, which was suggested to be involved in mate competition and spatial separation between males (20). Given that olfactory communication seems to be of greater importance in male koalas than female koalas, the lower olfactory bulb to brain weight proportion in our study could be attributed to the sex of our koala being female. In order to determine if olfactory bulb proportion is related to scent marking, a comparison of olfactory bulb to brain weight proportion should be conducted between male and female koalas.

#### 4.2.3 Cerebral hemispheres

On T1/T2*weighted MRI images, gray matter has a higher signal intensity while white matter has lower signal intensity. The ovoid shape of the cerebral hemispheres is similar to the *Vombatidae*, *Petauridae* and *Phalangeridae* brains (4).

The volume of the cerebral hemispheres in this study is approximately 9336.92 mm^3^ (Table 1), which is 59.39% of the total brain volume. The proportion of the brain volume taken up by the cerebral hemispheres is between the gyrencephalic brains of large macropods whereby the isocortex takes up 73% of total cortex and the lissencephalic brains of small dasyurids from the order of dasyuromorphia, whereby the isocortex takes up 50–60% of total cortex (21). The lissencephaly of the koala cortex was suggested to be a consequence of the low number of cortical neurons (21).

The koala cerebral white matter volume is 1495.47 mm^3^ (Table 1), which is 9.51% of the total brain volume. A study investigating how white matter volume and white/gray matter ratio in mammalian species were influenced by cortical folding produced a model which could predict gray matter, white matter and folded surface properties across all mammalian species without neuron-number-based optimization, as long as cortical thickness and cortical volume or exposed cortical surface area was known (22). Mota et al. (22) postulated that regardless of the number of neurons, the folded conformation of the cortex which is the most energetically favorable determines the relative white matter volume Given that the gray matter volume in koalas is unexpectedly large for the low gyrification index, it may be a good species candidate for testing the validity of this model.

##### 4.2.3.1 Interhemispheric connection

It has been known that marsupials lack a corpus callosum and that koalas and kangaroos in particular possess the fasciculus aberrans, which is an additional axonal tract connecting the dorsal and ventral aspects of the anterior commissure via the internal capsule (12).

In the koala, it was determined that temporal isocortical commissural connections pass through the external capsule to reach the anterior commissure, while the parietal isocortical commissural connection passes through the fiber bundle from the internal capsule through the fasciculus aberrans to the anterior commissure (23).

##### 4.2.3.2 Hippocampus

In chimpanzees with a mean whole brain volume of 379,740 mm^3^, the mean hippocampal volume was 2264.71 mm^3^, which was 0.6% (24). In primates, social group size was the main predictor of total hippocampal size and was postulated to be due to involvement in social memory (25). The koala hippocampus has a volume of 370.81 mm^3^ (Table 1), which is 2.36% of total brain volume. The hippocampus in chimpanzees takes up a smaller proportion of total brain volume than the koala, which supports the solitary nature of the koala since gene–environment interactions have been linked to most neural patterns and features (26, 27). The hippocampus takes up a smaller percentage of the brain in koalas as compared to the wombat, whose hippocampus takes up 5.9% of the brain (28). In the study, the large hippocampus of the wombat was attributed to the wombat’s need to navigate through their extensive burrow. Since koalas typically spend most of their day resting and often rely on scent to maintain their territory, they may not require the formation of complex mental maps like the wombat does.

The hippocampus of male non-breeding rodents has been found to be larger than male breeding rodents, breeding female rodents and non-breeding female rodents due to endogenous testosterone levels (29). This supports seasonal differences in the hippocampal volume in species which demonstrate spatially demanding behaviors during specific seasons, which is applicable in koalas which move around to mate during the breeding season.

#### 4.2.4 Putamen and caudate nucleus

The putamen and the caudate nucleus are primarily involved in the regulation of movement planning and execution as well as supporting learning, with secondary involvement in language and emotional processing (30). According to a study on putamen volume, normal humans have a putamen with an average volume of 7,140 mm^3^ (31). Based on existing literature, the human brain has an average volume of 1,330,000 mm^3^ (32). With this knowledge, we can determine that the putamen takes up approximately 0.5% of the total brain volume. According to the study on volume of caudate nucleus, normal humans have a caudate nucleus with an average volume of 2,730 mm^3^, which takes up approximately 0.2% of the total brain volume (31). The higher proportion of brain volume of the putamen in humans complements the fact that humans practice fine motor control and exist in complex social groups with higher demands on learning and emotional processing.

The koala putamen has a volume of 30.38 mm^3^ (Table 1), which is 0.19% of total brain volume. The koala caudate nucleus has a volume of 110.70 mm^3^ (Table 1), which is 0.7% of the total brain volume. The koala putamen and caudate nucleus to total brain volume ratio, respectively, are lower than in humans, which suggests simpler emotional processing and learning systems in koalas, as well as the absence or more primitive form of communication not to the complexity of language as in humans.

In primates and humans, it has been found that the volume of the caudate is roughly 75% the size of the putamen (31). However, in koalas, the volume of the caudate is 367% of the size of the putamen. Sea lions and coyotes also have a greater caudate volume than putamen volume and this was attributed to the selection for goal directed predation which is governed by the caudate nucleus, over manual dexterity and complex motor learning which is governed by the putamen (33). In koalas, this finding could suggest that goal directed behavior such as finding suitable eucalyptus trees are prioritized over fine motor control.

#### 4.2.5 Thalamus

The thalamus was segmented with reference to the labeled stained coronal sections of the brown antechinus (*Antechinus stuartii*) (4). The volume of the left and right thalamus was approximately 412.84 mm^3^ (Table 1), which is 2.62% of the total brain volume. In humans, the volume of both the right and left thalamus is approximately 13,600 mm^3^, which is 1% of the total brain volume (34). Comparing the koala and humans, the koala’s thalamus takes up a larger proportion of its total brain volume. However, when compared to the wallaroo, wombat and the eastern gray kangaroo whose thalamus takes up 4.68%, 5.29% and 6.08% of the total brain volume respectively, the koala has a comparatively smaller thalamus (28, 35, 36).

The role of the thalamus is to regulate and relay information between the basal nuclei and cerebral cortex, and it is made up of different thalamic nuclei including the pulvinar nucleus and the ventroanterior/ventrolateral nuclei, which are involved in visuospatial attention and motor movements, respectively (37). Given that the koala is an arboreal creature and that they demonstrate a preference toward larger trees in the habitat due to the presence of more foliage and increased shade and safety as evidenced in Moore et al. (38), it can be postulated that the thalamus is of key importance in the survival of koalas and hence take up a larger percentage of brain volume than in humans. Given that the thalamus has a role in promoting wakefulness, the higher percentage volume of the thalamus in the wallaroo, wombat, and the eastern gray kangaroo may be related to the longer amount of time they remain awake as compared to the koala.

#### 4.2.6 Brainstem—midbrain, pons and medulla

Mapping the different sections of the brainstem on MRI was largely based on anatomical knowledge of relationships between structures, as opposed to observing differences in contrast or signal intensities between the midbrain, pons and medulla. Further differentiation of the major nuclei and tracts spanning across the brainstem usually requires other methods such as histology and/or tractography.

The volume of the koala midbrain was approximately 646.78 mm^3^ (Table 1), which is 4.11% of the total brain volume. In humans, the average volume of the midbrain was 4,030 mm^3^, which is 0.3% of the total brain volume (39). It was difficult to identify the dorsal and ventral tegmentum of the pons in this study due to the poor resolution and difficulty in differentiating the differences in signal intensity in this area. The volume of the koala pons was approximately 702.44 mm^3^ (Table 1), which is 4.47% of the total brain volume. In humans, the average volume of the pons was 10,400 mm^3^, which is 0.7% of the total brain volume (39). The volume of the koala medulla was approximately 525.51 mm^3^ (Table 1), which is 3.34% of the total brain volume. In humans, the average volume of the medulla was 3,800 mm^3^, which is 0.3% of the total brain volume (40).

In humans, the volume of the brainstem is known to take up 2.6% of the brain’s total weight (41). Overall, the midbrain, pons and medulla of koalas take up a higher percentage of the total brain volume as compared to humans. It has been established that when compared to increases in brain volume across species, the human cerebral cortex had expanded disproportionately, showing increased cortical folding (42). This could have resulted in a decreased proportion of the brainstem volume out of total brain volume, which on the inverse could explain the higher proportion of the brainstem volume out of total brain volume in the koala.

#### 4.2.7 Cerebellum

The cerebellar white matter is observed as the dark tracts within the cerebellum, with the cerebellar white matter tracts traversing across the vermis being the cerebellar commissure (Figure 2). In the Tammar Wallaby atlas, nine lobules of the cerebellar vermis were identified along with the fissures (4). In this study, while we are able to identify the vermis, there is difficulty in clearly identifying the fissures and folia which is supported by the observations made by Haight and Nelsons in 1987 of the poorly elaborated cerebellar hemispheres, reflecting poor cerebrocerebellar circuitry development.

Ashwell (43) described that diprotodontid metatherians have large cerebellar hemispheres compared to non-diprotodontids. Through visual comparisons of the MRI of the koala in this study to stained parasagittal sections through the cerebellum in a rat, cat, monkey and human, it can be observed that the koala more closely resembles cats in terms of the folding pattern of the cortex in the vermis and the cerebellar hemispheres (44). Intricacies in the folding pattern of the cerebellar cortex were linked with the folding of the cerebral cortex.

Maseko et al. (14) used a cerebellar quotient to demonstrate the relationship between brain mass and cerebellar volume. Using the same quotient, the cerebellar quotient of the koala is 1.08. This falls within the range recorded for primates, megachiropterans and insectivores and this single value indicates slight positive allometry in the relationship between brain mass and cerebellar volume. A larger sample size would be required to provide a more accurate representation of the cerebellar quotient in koalas and marsupials in general.

#### 4.2.8 Ventricles

The brain ventricular system in koalas is relatively similar to the ventricular system in eutherian mammals and thus humans, with the presence of the lateral ventricles, third ventricle, mesencephalic aqueduct and fourth ventricle.

The volume of the ventricles recorded in this study excludes some sections of the ventricular system which could not be segmented due to discontinuity of the ventricular space across slices. One of the possible reasons for this phenomenon could be collapse and shrinking of the ventricles, which is likely to occur with an extended period of post fixation ex-*situ* as mentioned in Maseko et al. (45). Research conducted on brain mass versus total ventricular volume on four groups of mammals, namely primates, megachiroptera, microchiroptera and insectivores, showed slight negative allometry in primates and positive allometry in megachiropterans, microchiropterans and insectivores (45). While there is no data specifically on marsupials, the work of Maseko et al. (45) investigating mammals suggests that there is a general positive allometric relationship between ventricular volume and brain mass - with exception of primates - meaning that we would expect a larger ventricular volume with heavier brain mass. Complete ventricular segmentation of various marsupial species will be required to determine whether koalas emulate this general observation.

## 5 Conclusion

This paper provides a foundation for creating a comprehensive koala brain atlas, but there are limitations to relying solely on differences in signal intensity and existing literature for brain segmentation of other marsupials and eutherians to create the adult koala brain atlas. In order to ensure high accuracy, other methods of brain mapping such as fiber tractography through diffusion tensor imaging should be employed. Histological sections of a complete koala brain paired with an MRI scan on the same specimen would be invaluable in building a brain atlas without any individual differences affecting interpretation. This study demonstrates that the pursuit of scientific knowledge, i.e., the brain mapping of endangered species, can be conducted with reduced environmental impact.

Additionally, it will be meaningful to create a brain atlas of koalas of different sexes and at different stages of development from neonates to pouch young and out of pouch joeys to further understand the development and evolution of the koala brain, which could aid us in relating structure to function.

## Data Availability

The original contributions presented in the study are publicly available. This data can be found here: https://url.au.m.mimecastprotect.com/s/5WIBCD1vlpTnBoNwRtWfNSjKy3-?domain=hdl.handle.net and https://doi.org/10.25910/qdcw-1k35.

## Footnotes

1
https://www.wava-amav.org/wava-documents.html
